# Lean Patients with Non-Alcoholic Fatty Liver Disease Have a Severe Histological Phenotype Similar to Obese Patients

**DOI:** 10.3390/jcm7120562

**Published:** 2018-12-17

**Authors:** Lukas Denkmayr, Alexandra Feldman, Lars Stechemesser, Sebastian K. Eder, Stephan Zandanell, Michael Schranz, Michael Strasser, Ursula Huber-Schönauer, Stephan Buch, Jochen Hampe, Bernhard Paulweber, Carolin Lackner, Heike Haufe, Karl Sotlar, Christian Datz, Elmar Aigner

**Affiliations:** 1 First Department of Medicine, Paracelsus Medical University, 5020 Salzburg, Austria; l.denkmayr@salk.at (L.D.); a.feldman@salk.at (A.F.); l.stechemesser@salk.at (L.S.); sebastian.eder@gmail.com (S.K.E.); s.zandanell@salk.at (S.Z.); m.schranz@salk.at (M.S.); m.strasser@salk.at (M.S.); B.Paulweber@salk.at (B.P.); 2 Obesity Research Unit, Paracelsus Medical University, 5020 Salzburg, Austria; c.datz@kh-oberndorf.at; 3 Department of Internal Medicine, General Hospital Oberndorf, Teaching Hospital of the Paracelsus Medical University Salzburg, 5110 Oberndorf, Austria; huber.schoenauer@gmail.com; 4 Department of Gastroenterology and Hepatology, Internal Medicine, University Hospital Dresden, 01307 Dresden, Germany; Stephan.buch@uniklinikum-dresden.de (S.B.); Jochen.Hampe@uniklinikum-dresden.de (J.H.); 5 Institute of Pathology, Medical University of Graz, 8010 Graz, Austria; karoline.lackner@medunigraz.at; 6 Institute of Pathology, Paracelsus Medical University, 5020 Salzburg, Austria; h.haufe@salk.at (H.H.); k.sotlar@salk.at (K.S.)

**Keywords:** fatty liver, non-alcoholic, obesity, insulin resistance, metabolic syndrome

## Abstract

A small proportion of lean patients develop non-alcoholic fatty liver disease (NAFLD). We aimed to report the histological picture of lean NAFLD in comparison to overweight and obese NAFLD patients. Biopsy and clinical data from 466 patients diagnosed with NAFLD were stratified to groups according to body mass index (BMI): lean (BMI ≤ 25.0 kg/m², *n* confirmed to be appropriate = 74), overweight (BMI > 25.0 ≤ 30.0 kg/m², *n* = 242) and obese (BMI > 30.0 kg/m², *n* = 150). Lean NAFLD patients had a higher rate of lobular inflammation compared to overweight patients (12/74; 16.2% vs. 19/242; 7.9%; *p* = 0.011) but were similar to obese patients (25/150; 16.7%). Ballooning was observed in fewer overweight patients (38/242; 15.7%) compared to lean (19/74; 25.7%; *p* = 0.014) and obese patients (38/150; 25.3%; *p* = 0.006). Overweight patients had a lower rate of portal and periportal fibrosis (32/242; 13.2%) than lean (19/74; 25.7%; *p* = 0.019) and obese patients (37/150; 24.7%; *p* = 0.016). The rate of cirrhosis was higher in lean patients (6/74; 8.1%) compared to overweight (4/242; 1.7%; *p* = 0.010) and obese patients (3/150; 2.0% *p* = 0.027). In total, 60/466; 12.9% patients were diagnosed with non-alcoholic steatohepatitis (NASH). The rate of NASH was higher in lean (14/74; 18.9% *p* = 0.01) and obese (26/150; 17.3%; *p* = 0.007) compared to overweight patients (20/242; 8.3%)). Among lean patients, fasting glucose, INR and use of thyroid hormone replacement therapy were independent predictors of NASH in a multivariate model. Lean NAFLD patients were characterized by a severe histological picture similar to obese patients but are more progressed compared to overweight patients. Fasting glucose, international normalized ratio (INR) and the use of thyroid hormone replacement may serve as indicators for NASH in lean patients.

## 1. Introduction

Non-alcoholic fatty liver disease (NAFLD) has become a severe socioeconomic burden in Western societies, affecting approximately one third of the population in most countries [1]. NAFLD is closely linked to the complications of excess adipose tissue on the population level; however, it has also been reported in lean patients with a body mass index (BMI) <25 kg/m² who are not considered to have excess adipose tissue [2,3]. According to the NHANES III (National Health and Nutrition Examination Survey) data, approximately 7% of lean individuals may have evidence of NAFLD. The risk of significant fibrosis was approximately one in four in the NHANES III NAFLD population [4,5].

The pathogenesis of NAFLD is multi-factorial and yet to be fully clarified. Most mechanisms for developing NAFLD are linked to changes in lipid and glucose homeostasis. In particular, insulin resistance plays a critical role in the development of NAFLD as it does in all other components of the metabolic syndrome, among which are obesity, type 2 diabetes mellitus (T2DM) and dyslipidemia [6]. The disease spectrum of NAFLD ranges from non-alcoholic fatty liver (NAFL) which is characterized by simple steatosis without inflammation or fibrosis to non-alcoholic steatohepatitis (NASH) that can progress further to cirrhosis, end-stage liver disease or hepatocellular carcinoma [7,8,9]. Obesity, age, advanced insulin resistance or T2DM have repeatedly been reported as risk factors for progression from NAFLD to NASH [5,10].

As lean patients, i.e., BMI ≤ 25.0 kg/m², lack obvious excess adipose tissue, differences in the disease mechanisms of NAFLD among lean compared to obese patients may exist. Available data in Caucasians suggest that lean NAFLD patients are characterised by impaired glucose metabolism, evidence of dysfunctional adipose tissue and a higher rate of carriage of the PNPLA3 minor allele [2]. Distinct clinical, biochemical and histological features have been observed in lean NAFLD subjects with proportionally increased visceral adiposity, low adiponectin, high pro-inflammatory cytokines and a lower proportion of NASH compared to overweight and obese subjects combined [11,12].

Since data on the histological picture of NALD in lean subjects are scarce, we aimed to provide a detailed report on the liver biopsy results of lean compared to overweight and obese NAFLD patients. 

## 2. Material and Methods

### 2.1. Study Population

The study population consisted of all consecutive patients referred to our clinics that had finally received the clinical and histological diagnosis NAFLD between 1997 and 2016. In total, 466 patients with a diagnosis of NAFLD were included in our study. The study cohort consisted of 329 males and 137 females aged between 18 and 75 years. 

Data were collected over the years as patients were seen in the liver outpatient clinics and data were analysed retrospectively. The study was approved by the local ethics committee (Ethikkommission des Landes Salzburg). 

### 2.2. Clinical and Laboratory Evaluation

Subjects were referred to our liver outpatient clinic for the work-up of unexplained elevation of liver tests. All study patients underwent a clinical examination at the time of visit of the liver outpatient clinics and at the time of biopsy. None of the patients showed signs of cardiac or renal insufficiency, infectious diseases or systemic autoimmune disorders. After an overnight fast, venous blood was drawn for the determination of laboratory parameters. These included liver tests, lipids, C-reactive protein, erythrocyte sedimentation rate, serum iron parameters, copper, ceruloplasmin and fasting glucose. The blood drawing was performed within a month of the liver biopsy. Thus, underlying infectious, autoimmune or hereditary etiologies of liver diseases were ruled out. Anti-nuclear antibody testing was done in all subjects with a cut-off titer of 1:160. Subsets including anti-mitochondrial (AMA), liver-kidney microsome (LKM), liver cytosol (LC), soluble liver antigen (SLA), and anti-smooth muscle antibodies (ASMA) were tested. At antibody positivity of 1:160 without histological changes compatible with autoimmune etiology but of NAFLD, subjects were diagnosed as NAFLD. The amount of alcohol intake was assessed by history taking and in case of reliable information on the amount of alcohol consumption (<40 g/day in males, <20 g/day in females) the etiology of liver disease was judged to be non-alcoholic. To determine the presence of metabolic syndrome, the ATPIII Criteria for metabolic syndrome were applied [13]. Diabetes was diagnosed when patients were on antidiabetic medication, had a fasting glucose level above 126 mg/dL or HbA1c (glycosylated hemoglobin) was 6.5% or higher. Thyroid hormone replacement at the time of liver biopsy was recorded.

Additionally, the following single nucleotide polymorphisms (SNPs) were analysed in patients where material was available: TM6SF2 *(rs58542926)*, MBOAT7 *(rs641738)*, PNPLA3 *(rs738409)* and SERPINA1 (*rs28929474*, genetic locus of alpha-1-antitrypsin, A1AT) [14]. All genetic studies were only performed later in some subjects where frozen full blood had been available for other investigations and these data had been collected over time. Hence, genetic studies were not performed systematically. The genetic data presented are all data attainable in this population.

The non-invasive scores for estimation of NAFLD disease severity NAFLD fibrosis score (NFS) and Fib4 were calculated [15,16].

### 2.3. Liver Biopsy and Histological Examination of Liver Biopsy Samples 

The indication to conduct a liver biopsy was made by the treating physician. Liver biopsies were performed mostly due to persistently elevated aminotransferase levels (for 3–6 months) and/or the presence of fatty liver detected by imaging and/or risk factors for advanced disease (e.g., metabolic syndrome, age >45 years, obesity, diabetes) and/or suspected advanced fibrosis or cirrhosis as determined by abnormal laboratory (low platelet count) and imaging (US, CT or MRI) tests. However, if unequivocal clinical or laboratory signs of liver cirrhosis were present a liver biopsy was not performed. In our clinics, the clinical standard for liver biopsies is to obtain one or two specimen at the liver biopsy depending on the length of the first biopsy. At least 15–18 mm of liver tissue is used for histological analysis corresponding to a sufficient number of portal tracts.

All liver biopsy samples were re-analysed in 2017 for current criteria relevant for diagnosing, grading and staging NAFLD as detailed below. Dewaxed sections (4 µm) of each case were processed according to routine protocols and stained with hematoxylin and eosin and Mallory trichrome (connective tissue stain). All slides were evaluated by two pathologists unaware of the clinical data in consensus using a multiheaded microscope. Histological grading and staging of NAFLD components were performed as published by Kleiner et al. [17] by application of numerical scores. Accordingly, separate scores were reported for steatosis (0–3), lobular inflammation (0–3), hepatocellular ballooning (0–2), Mallory-Denk bodies (0–2), and portal inflammation (0–1). Fibrosis stage was assessed on a 5-step scale including stages 0 (none), 1 (centrilobular or pericellular), 2 (centrilobular plus periportal), 3 (bridging), and 4 (cirrhosis). Ballooned hepatocytes were characterized by a marked increase in size (approximately two times the size of a normal hepatocyte), rounded cell shape and pale staining of the cytoplasm. 

The presence of steatosis (>5% of the parenchyma), lobular inflammation and ballooning was required for diagnosis of NASH [18]. The diagnostic algorithm for histological classification as NAFLD or NASH was followed as reported by Bedossa et al. in the SAF score. [19] The constellation of steatosis with fibrosis ≥2 and lobular inflammation without ballooning was found in 2 lean subjects, in 2 overweight and 3 obese subjects. These subjects did not qualify as NASH according to the SAF algorithm. However, in the context of all clinical, biochemical and histological data available, these subjects were counted as NASH and not NAFL [20,21]. The clinical history, laboratory and the histological results were compatible with the diagnosis of NAFLD. Study participants were stratified to one of three groups according to the individual’s BMI. Patients with a BMI ≤ 25.0 kg/m² were considered as “lean” (*n* = 74), those with a BMI > 25.0 and ≤ 30.0 kg/m² as “overweight” (*n* = 242) and patients with a BMI ≥ 30.0 kg/m² were as “obese” (*n* = 150).

### 2.4. Statistical Analysis 

Statistical analysis was performed using SPSS Statistics (IBM Corp. Released 2013, IBM SPSS Statistics for Windows, Version 22.0, Armonk, NY, USA). Subject characteristics are expressed as means and standard deviations for normally distributed variables and were analyzed using analysis of variance (ANOVA) and by Kruskal-Wallis test in case of not normal distribution. Categorical variables, including prevalence of genetic polymorphisms, are reported as frequencies and were compared using chi-square analysis. A two-sided p-value of less than 0.05 indicated statistical significance. *We then aimed to elucidate the particularity of the risk for NASH in the subgroup of lean subjects.* To calculate parameters linked to NASH in lean subjects, univariate logistic regression analysis was performed. Variables that showed significant difference in the univariate model were included in a multiple logistic regression analysis. Model calibration was determined through forward stepwise variable selection using the likelihood ratio algorithm while age and sex were forced in the model for adjustment.

## 3. Results 

### 3.1. Clinical and Biochemical Characteristics 

The study groups were similar with regard to sex and age distribution. Obese subject had more components of the MetS biochemically and anthropometrically. The clinical and biochemical details of the study groups are summarized in Table 1.

### 3.2. Genetic Variants 

Due to the retrospective design of the study, genetic variants are reported which had been determined previously as part of other studies (TM6SF2, PNPLA3, MBOAT7) or the clinical routine (A1AT) where available, rendering only incomplete and varying data sets. No group differences were observed for the well documented NAFLD risk alleles PNPLA3 and TM6SF2; however, A1AT risk alleles were only observed in the obese and lean but not in the overweight cohort. For group comparisons, heterozygous and homozygous carries of the risk allele were counted together, the details are reported in Table 2.

### 3.3. Histological Results 

The details of the histological analysis are summarized in Table 3.

**Steatosis:** The degree of steatosis was 26.1 ± 18.8% in lean, 28.4 ± 19.8% in overweight and 36.3 ± 20.5% in obese patients which was similar between lean and overweight patients but higher in obese patients compared to lean and overweight.

### 3.4. Disease Activity 

The lean NAFLD group had a higher proportion of patients with lobular inflammation compared to the overweight or obese NAFLD study groups (*p* < 0.001) while the two other groups were similar. The rate of patients with portal inflammation was similar between groups. Assessment of hepatocellular ballooning revealed a statistically significant difference between the different BMI categories (*p* < 0.001). The overweight group had the lowest proportion of patients with hepatocellular ballooning, while lean patients had the highest. 

### 3.5. Fibrosis 

The rates of all fibrosis subgroups were similar between the OB and the lean group while the overweight group had a significantly less pronounced picture. The prevalence of histologically detected cirrhosis was higher in the lean study group with 6 cirrhotic patients (8.1%) compared to the overweight (4; 1.7%, *p* = 0.010) or obese patients (3; 2.0%; *p* = 0.027). 

### 3.6. NASH

**Figure 1:**
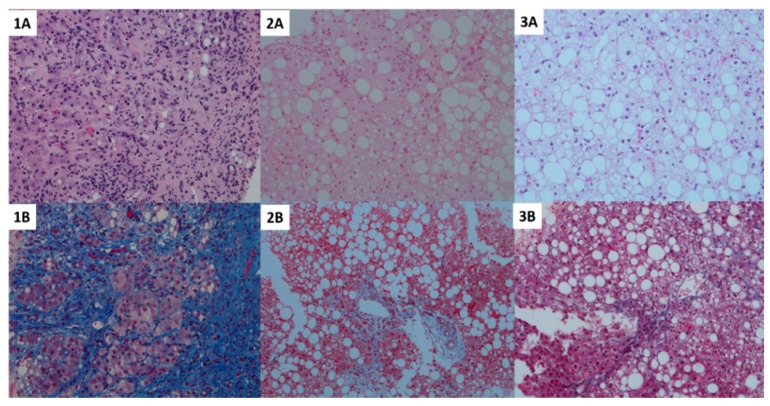
Histological pictures indicative of the statistical results including hematoxylin and eosin staining (top row) and CAB staining for fibrosis (bottom row) of the 3 groups stratified by BMI. Panel **1A** and **1B**—lean; **2A** and **2B**—overweight; **3A** and **3B**—obese group. A higher degree of fibrosis with together with a lower degree of steatosis was observed in lean subjects (left, **1A** and **1B**), while more fat accumulation but lower fibrosis and disease activity was observed in overweight subjects (middle, **2A** and **2B**). Obese subjects presented with the highest degree of fibrosis and a fibrosis and NASH similar to lean subjects (right, **3A** and **3B**).

In total, 60 patients were classified as NASH. Reflecting the various histological components of the disease spectrum, the rate of NASH was lower in overweight patients compared to the two other groups (Figure 1).

### 3.7. Identification of Non-Invasive Indicators for NASH in Lean Patients Appropriate as is Here

We then aimed to identify non-invasive predictors of NASH in lean patients. Age, fasting glucose, the presence of MetS, use of thyroid hormone replacement, hemoglobin, INR, gamma-GT and AST were associated with NASH in univariate regression analysis. Further, a multivariate model including age and sex was developed with a forward stepwise inclusion of parameters identified in the univariate analysis as given in Table 4. Fasting glucose, INR and the use of thyroid hormone replacement were identified as independent predictors of NASH in lean patients. Excluding thyroid hormone replacement from the model, which may carry a gender bias, from the model did not significantly alter results (B) in Table 4. The absolute numbers of these three parameters in the lean NAFL subjects in comparison to the lean NASH group are reported in Table 5.

## 4. Discussion

We herein report details on the histological disease spectrum of NAFLD in lean patients. In summary, we found that lean NAFLD patients presented fewer components of metabolic syndrome compared to obese and overweight patients with NAFLD. Histologically, however, lean NAFLD patients had a high degree of inflammation, ballooning, and fibrosis, and therefore a high proportion of subjects with NASH [4,22,23].

We have reported in an independent investigation without liver biopsies that lean patients with NAFLD had a higher degree of visceral adipose tissue and an adverse adipokine profile compared to lean healthy subjects which indicated a pronounced degree of adipose tissue dysfunction even in the absence of obesity in these patients [2]. However, data on the histological disease manifestations in lean NAFLD patients are limited. 

Overall, we detected a low proportion of NASH subjects in our cohort. We attribute this to the clinical practice over long periods when liver biopsies were conducted mainly due to persistently elevated liver tests irrespective of the true risk of having NASH. The prevalence of NASH in our study cohort was comparable to a similarly recruited cohort reported by Williams et al. [24]. This has changed recently with the availability of reliable tools for non-invasive testing where a high proportion of subjects undergoing biopsy according to predefined non-invasive biochemical and liver stiffness measurement criteria are then confirmed to have NASH histologically. 

Several aspects of our findings add notable information to the field of NAFLD. First, the lower degree of hepatic steatosis in lean subjects was unrelated to the high degree of inflammation, hepatocellular ballooning, fibrosis and consequently the diagnosis NASH in these patients. In contrast to our findings, it has recently been reported from an Asian population that lean patients had a lower degree of histological severity and also lower mortality compared to obese patients [25]. Similarly, a recent meta-analysis including 4 Asian and 4 Caucasian cohorts on histological severity of 493 lean compared to 2209 overweight/obese subjects suggested a lower degree of histological severity in the lean group [26]. However, in that analysis overweight and obese subjects were counted as one group which may be important to distinguish. Additionally, the same BMI cut-off of 25 kg/m² was used in Asian and Caucasian subjects. In Asians a BMI of 23 kg/m² may correspond to 25 kg/m² in Caucasians and 25 to 30 kg/m² in Caucasians [27]. In another study among Caucasians, Akyuz et al. compared the histological severity of lean to overweight and obese subject combined.12 Besides this combined analysis of overweight and obese subjects, lean subjects were significantly younger and comprised only 7.6% of the study population in that study which may relate to the difference to our findings where all three groups were of similar age. Importantly, in support of our data, a high rate of mortality and morbidity has recently been reported in lean NAFLD subjects in a Caucasian population [28]. 

The high rate of NASH in lean subjects indicates that a fatty liver in a lean subject may serve as a marker of individuals that are particularly susceptible to liver injury in response to even a lower degree of systemic metabolic alterations. Thus, our findings strongly suggest that the underlying pathophysiology between NAFLD in lean patients and typical obesity-associated NAFLD may differ. 

We have recently reported that lean NAFLD patients had a higher rate of the PNPLA3 risk allele^2^. In the present study, we found that lean patients had a higher rate of the A1AT risk allele and only a trend toward significance for the carrier rate of the PNPLA3 risk allele. Carriage of the A1AT has recently been reported as a risk allele in NAFLD. Similarly, A1AT has been identified as a risk factor for progression in several other liver diseases like hemochromatosis or hepatitis C [14,29,30]. Our data support that A1AT may have a role particularly in the context of lean NAFLD although the absolute numbers of carriers of the risk alleles were low. 

The biochemical markers of higher AST and GGT levels in the clinical routine increase the suspicion of alcohol as a causative agent of fatty liver disease. Although the patients’ history was carefully taken and documented, every clinician is well aware of the snares of intentional underreporting of the amount of alcohol consumed. Moreover, this would hold true for all three groups in a similar fashion. From another perspective, these same data may be interpreted as a particular individual susceptibility to lower amounts of alcohol as a liver toxin in these patients analogous to the lower degree of systemic metabolic alterations in these subjects. Nevertheless, our data highlight that the role of alcohol consumption in NAFLD may still require further study as its role may vary in different patient groups. 

Although our study cohort represented all consecutive subjects that had received the diagnosis of NAFLD over the indicated time period, the process of subject selection for liver biopsy may be biased due to unidentified in-between-group differences of underlying causes for NAFLD as suggested from different levels of biliary tree enzymes in the various groups.

Another mechanism of disease that will merit particular attention in lean subjects is the role of the intestinal microbiome. Due to the lower amount of total excess adipose tissue, but previously reported severe visceral adipose tissue dysfunction, intestinal microbial products owing to their anatomical proximity appear as plausible players in the concert of factors that result in liver inflammation and fibrosis in the absence of obesity [31]. Additionally, less explored factors such as sarcopenia will need to be studied in-depth in the future [32].

The surprisingly high degree of liver damage additionally indicates that the natural course of lean NAFLD with regard to causes of death and morbidity will need to be studied further. We have recently started to evaluate these clinically relevant endpoints in our cohort and have preliminary results that pronounced liver histological findings will translate into clinically important endpoints over time. Our data also suggest that commonly used non-invasive scores may perform significantly different across BMI strata depending on the weight BMI is given in the calculation [33]. Fib4 reflected the histological extent of fibrosis well while the BMI-based NFS yielded inappropriately low risk for lean subjects. It is a limitation of our study that detailed mechanisms of disease such as underlying causes of insulin resistance and defective adipose tissue expansion could not be studied. These may well be different in the subgroups of this population; however, we are only able to speculate on this topic due to the lack of data. Additionally, the analysis of reported biomarkers such as cytokeratin 18, hyaluronic acid, type 4 collagen, and Mac-2 binding protein glycosylated isomers would be informative in this population.

Several laboratory and clinical parameters were different between lean patients with NASH compared to NAFL. Among these are fasting blood glucose, clinically diagnosed T2DM, higher AST and GGT levels and also the current use of thyroid hormone replacement therapy. While higher glucose concentrations may indicate the role of insulin resistance as the key mechanism that underlies NASH, AST and GGT may rather be a marker of injury than an indicator of underlying causes. Of note, TSH levels were not different between these groups which renders it unlikely that insufficient replacement therapy is the true culprit underlying progressive liver damage. Hypothyroidism itself can be considered a risk factor for either developing NAFLD or for the progression of the disease [34]. We propose two potential explanations for the observed associations—either the effect of thyroid hormone replacement therapy on liver metabolism is lower compared to the estimated effect suggested by normal TSH concentrations or a common immunological mechanism that is not detected by serum autoantibodies or liver biopsy plays an additional role particularly in lean subjects.

Although the absolute number of subjects with NASH was low, resulting in very high standard deviations in the regression model for thyroid hormone replacement, these findings suggest that the link between immunological mechanisms, sex and NAFLD should be further investigated, particularly in lean subjects with NAFLD. Recently, the role of thyroid hormones in the development of NAFLD was further elucidated by reporting a complex interplay between the liver and adipose tissue, particularly in mildly hypothyroid mice, confirming that further studies in humans will need to be done [35]. 

In conclusion, these results suggest that NAFLD in lean patients presents with a severe histological picture and should be further investigated both clinically and with regard to potential distinct mechanisms of disease. Indicators such as elevated fasting glucose, any increase in INR, or the use of thyroid hormone replacement should particularly raise the clinical suspicion for NASH in lean subjects.

## Figures and Tables

**Table 1:** Clinical, anthropometrical and biochemical characteristics of the study population.

Clinical Characteristics	Lean NAFLD (*n* = 74)	Overweight NAFLD (*n* = 242)	Obese NAFLD (*n* = 150)	*p*-Value	Lean vs. Overweight	Lean vs. Obese ^2^	Overweight vs. Obese ^3^
Age (years)	48.7 ± 14.8	49.6 ± 13.8	50.4 ± 12.3	0.122	*	*	*
Sex (M/F)	43/31	179/63	107/43	0.675	*	*	*
Systolic BP (mm Hg)	120.5 ± 16.7	127.5 ± 18.2	135.1 ± 20.6	0.001	0.163	0.001	0.021
Diastolic BP (mm Hg)	75.0 ± 10.7	78.7 ± 11.3	83.4 ± 12.5	0.001	0.287	0.001	0.018
Hypertension (y/n)	19/55 (25.6%)	86/156 (35.5%)	92/58 (61.3%)	<0.001	0.360	<0.001	<0.001
BMI (kg/m²)	23.1 ± 1.5	27.4 ± 1.5	33.1 ± 2.9	<0.001	<0.001	<0.001	<0.001
Diabetes (y/n)	13/61 (17.5%)	67/175 (27.6%)	68/82 (45.3%)	<0.001	0.277	<0.001	<0.001
MetS (y/n)	10/64 (13.51%)	73/169 (30.17%)	105/45 (70%)	<0.001	0.016	<0.001	<0.001
Components of MetS	1.08 ± 1.03	1.57 ± 1.18	2.80 ± 1.11	<0.001	0.004	<0.001	<0.001
TG (mg/dL)	186.3 ± 146	179.1 ± 112.2	213 ± 220.6	0.131	*	*	*
High TG (%)	34 (45.9%)	115 (42.0%)	80 (53.3%)	0.315	*	*	*
Cholesterol (mg/dL)	218.7 ± 58.1	216.5 ± 50.3	219.2 ± 51.3	0.873	*	*	*
HDL-C (mg/dL)	58.1 ± 25.22	49.61 ± 14.70	49.12 ± 15.3	<0.001	<0.001	<0.001	1.000
Low HDL-C (%)	22 (29.7%)	105 (38.3%)	64 (42.7%)	0.493	*	*	*
LDL-C (mg/dL)	127.3 ± 50.6	137 ± 42.6	136.0 ± 42.9	0.243	*	*	*
Fasting Glucose (mg/dL)	94.5 ± 22.7	105.9 ± 31.8	113.6 ± 37.0	<0.001	0.030	<0.001	0.079
HbA1c (mmol/mol)	6.1 ± 0.9	6.3 ± 1.0	6.5 ± 0.7	0.077	*	*	*
Bilirubin (mg/dL)	1.3 ± 0.5	1.5 ± 0.7	1.1 ± 0.4	0.325	*	*	*
GGT (IU/L)	191.3 ± 119	128.2 ± 127.4	196 ± 222.2	0.006	0.101	0.879	0.011
AST (IU/L)	53.7 ± 41.9	43.0 ± 26.9	53.8 ± 40.8	0.004	0.060	0.985	0.009
ALT (IU/L)	60.0 ± 36.4	66.9 ± 48.5	87.0 ± 63.7	<0.001	0.961	0.001	0.001
AP (IU/L)	116.2±100	92.7 ± 65.6	92.3 ± 58.3	0.031	0.037	0.052	1.000
Hemoglobin (g/dL)	14.6 ± 1.6	15.3 ± 1.4	15.4 ± 1.2	<0.001	0.001	<0.001	1.000
Platelets (G/L)	218.2 ± 69.0	218.5 ± 60.0	211.2 ± 57.0	0.491	*	*	*
Ferritin (µg/L)	500.5 ± 544	472.2 ± 443.9	597.2 ± 534	0.054	*	*	*
Transferrin Sat. (%)	36.5 ± 31.8	33.2 ± 14.1	34.0 ± 13.6	0.430	*	*	*
Thyroid hormone replacement (y/n) [m/f]	10/64 (13.5%) [2;8/40;24]	25/217 (10.3%) [9;16/172;45]	24/126 (16.0%) [13;11/93;3]	0.210	*	*	*
Fib4	2.05 ± 2.28	1.41 ± 1.11	1.61 ± 1.54	0.006	0.004	0.111	0.633
NAFLD fibrosis score	−1.64 ± 2.06	−1.19 ± 2.16	−0.07 ± 2.13	<0.001	0.99	0.001	<0.001

**^1^***p*-value indicating the level of significance between lean and overweight patients; **^2^***p*-value indicating the level of significance between lean and obese patients; **^3^***p*-value indicates the level of significance between obese and overweight patients. Abbreviations: OW—overweight, BMI—Body Mass Index; BP—blood pressure; MetS—Metabolic syndrome. Abbreviations: ALT—alanine aminotransferase; AP—alkaline phosphatase; AST—aspartate aminotransferase; GGT—gamma glutamyltranspeptidase; HbA1c—hemoglobin A1c; HDL-C—high density lipoprotein cholesterol; LDL—low density lipoprotein cholesterol; OW—overweight; TG—triglycerides; * denote calculations not performed due to insignificant difference in the comparison of all groups.

**Table 2:** Carrier rates of genes determined; where available from previous data collections, genotypes of determined alleles are reported. Due to the retrospective nature of the study only incomplete sets were available, carriers of at least one risk allele were counted as one group.

Genotype	Lean	Overweight	Obese
TM6SF2 CC/CT, TT (%)	36/10 (78.3/21.9)	114/32 (80.3/19.7)	71/14 (83.5/16.5)
PNPLA3 CC/CG, GG (%)	15/35 (30.0/70.0)	64/104 (38.1/61.9)	25/68 (26.9/73.1)
A1AT CC/CT, TT (%)	43/3 (93.5/6.5) *	143/0 (100/0)	78/6 (92.9/7.1) **
MBOAT7 CC/CT, TT (%)	2/4 (33.3/66.7)	7/18 (28.0/72.0)	7/12 (36.8/63.2)

* *p*-value indicating significant difference *p* < 0.05 between overweight NAFLD and lean or obese study patients as calculated by chi-square analysis, ** indicates *p* < 0.001 compared to overweight subjects.

**Table 3:** Summary of the histological characteristics of the study population.

Histological Parameter	Lean	OW	OB	*p*-Value	Lean vs. Overweight	Lean vs. Obese	Overweight vs. Obese
	*n* = 74	*n* = 242	*n* = 150				
Steatosis [%]	26.1 ± 18.8	28.4 ± 19.8	36.3 ± 20.5	<0.001	0.379	<0.001	<0.001
Steatosis Degree (1/2/3)	47/25/2	147/86/9	63/76/11	0.002	0.866	0.008	0.001
Portal Inflammation (>0)	11 (14.9%)	21 (8.7%)	19 (13.3%)	0.138	*	*	*
Iobular Inflammation (>0)	12 (16.2%)	19 (7.8%)	25 (16.7%)	<0.001	0.011	0.891	0.066
Ballooning (>0)	19 (25.7%)	38 (15.7%)	38 (25.4%)	0.001	0.014	0.985	0.006
Perisinusoidal Fibrosis (>0)	22 (29.7%)	47 (18.7%)	44 (28.3%)	0.034	0.051	0.857	0.078
Periportal Fibrosis (>0)	19 (25.7%)	32 (13.3%)	37 (24.7%)	0.010	0.019	0.731	0.016
Bridging Fibrosis (>0)	10 (13.6%)	17 (7.4%)	15 (10%)	0.202	*	*	*
Cirrhosis	6 (8.1%)	4 (1.7%)	3 (2%)	0.010	0.010	0.027	1.000
NASH	14 (18.9%)	20 (8.3%)	26 (17.3%)	0.008	0.049	1.000	0.027

Abbreviations: infl.—inflammation; *p*—level of significance calculated by chi-square analysis regarding the rate of the respective parameter. * denote calculations not performed due to insignificant difference in the calculation of overall difference between groups.

**Table 4:** Multivariate regression model of parameters significantly related to NASH (**A**) and excluding thyroid hormone replacement which may carry a sex bias (**B**).

	*p*-Value	OR (95% CI)
**(A)**		
Age	0.893	1.007 (0.91–1.12)
Sex	0.081	7.400 (0.78–70.29)
Fasting glucose	0.024	1.061 (1.01–1.12)
INR	0.152	0.995 (0.99–1.00)
Thyroid replacement	0.007	31.731 (2.54–396.96)
Constant		
**(B)**		
Age	0.341	1.042 (0.958–1.133)
Sex (male)	0.429	2.340 (0.285–19.227)
Fasting Glucose	0.095	1.042 (0.993–1.094)
INR	0.010	0.868 (0.778–0.967)
Constant		

**Table 5:** Comparison of clinical and biochemical characteristics identified to be predictors for NASH in lean patients between lean NAFL and lean NASH patients in the multivariate model.

Parameter	Lean NAFL (*n* = 57)	Lean NASH (*n* = 14)	*p*-Value
Fasting glucose (mg/dL)	89.6 ± 18.7	114.8 ± 26.9	0.004
INR	1.0 ± 0.1	1.2 ± 0.2	0.002
Thyroid replacement yes (%)	3 (5.3%)	7 (50%)	<0.001
